# SARS-CoV-2 infection in HIV-infected patients: potential role in the high mutational load of the Omicron variant emerging in South Africa

**DOI:** 10.1007/s11357-022-00603-6

**Published:** 2022-06-24

**Authors:** Katalin Réka Tarcsai, Oliga Corolciuc, Attila Tordai, József Ongrádi

**Affiliations:** 1 Doctoral School, Semmelweis University, Budapest, Hungary; 2 Department of Transfusion Medicine, Semmelweis University, Nagyvárad tér 4, Budapest, 1089 Hungary; 3 Institute of Public Health, Semmelweis University, Budapest, Hungary

**Keywords:** Coronavirus, Variant Omicron, Human immunodeficiency virus, Interaction, Exonuclease, Divalent cations

## Abstract

A new variant of SARS-CoV-2 named Omicron (B.1.1.529) was isolated from an HIV-infected patient in Botswana, South Africa, in November 2021. Whole genome sequencing revealed a multitude of mutations and its relationship to the mutation-rich Alpha variant that had been isolated from a cancer patient. It is conceivable that very high prevalence of HIV-infected individuals as susceptible hosts in South Africa and their immunocompromised state may predispose for accumulation of coronavirus mutations. Coronaviruses uniquely code for an N-terminal 3′ to 5′exonuclease (ExoN, nsp14) that removes mismatched nucleotides paired by the RNA dependent RNA polymerase. Its activity depends preferably on Mg^2+^ and other divalent cations (manganese, cobalt and zinc). On the contrary, methyl transferase activity of non-structural protein (nsp) 14 and nsp16 both complexed with nsp10 requires Mn^2+^. Enzymes in successive stages of HIV infections require the same cations. In HIV-infected organisms, a subsequent coronavirus infection encounters with altered homeostasis of the body including relative starvation of divalent cations induced by interleukin production of HIV-infected cells. It is hypothesized that selective diminished efficacy of ExoN in the absence of sufficient amount of magnesium may result in the accumulation of mutations. Unusual mutations and recombinations of heterologous viruses detected in AIDS patients also suggest that long-lasting persistence of superinfecting viruses may also contribute to the selection of genetic variants. Non-nucleoside reverse transcriptase inhibitors partially restore divalent cations’ equilibrium. As a practical approach, implementation of highly active antiretroviral therapy against HIV replication and vaccination against coronaviruses may be a successful strategy to reduce the risk of selection of similar mutants.

## Emergence of the SARS-CoV-2 Omicron variant

Almost two and half years since the first reported cases of SARS-CoV-2 recognized, a new variant named Omicron emerged in South Africa and rapidly spread all over the world. Its characteristics shocked both experts and the public [1–3]. Analysis of stored specimens showed that the earliest case was detected on November 9, 2021, but one cannot exclude that unidentified cases were missed before. Following exceptionally rapid identification, the first genome sequence was submitted on November 23, subsequently on November 26, WHO designated Omicron (B.1.1.529) as a variant of concern (VoC) [1, 4] Since that, genomes of several isolates have been sequenced; subsequently, these were grouped into three lineages or subvariants: BA1 (B.1.1.529.1), BA.2 (B.1.1.529.2), and BA.3 (B.1.1.529.3). (At time of writing, retrospective analysis discovered new subvariants in Botswana and SA: BA.4 and BA.5. They share many of the same mutations with BA.1 and BA.2, but they have distinct ones. They have spread to other continents and are circulating at low levels, but they might induce a new wave of infection in SA [32].) Omicron BA.1 contains 60 mutations as compared to the first SARC-CoV isolate obtained in China; from these, 32 mutations are in the spike (S) region suggesting its role for the profoundly altered pathogenicity in humans [1, 3]. The majority are point mutations resulting in amino acid exchange. Furthermore, a cluster of short deletions and an insertion in the NTD region of S gene, as well as randomly distributed three other deletions in the nsp3 (papain-like protease) and nsp5 (chymotrypsin-like protease) and structural protein N gene, have been identified. [2, 5]. Several of mutations overlap with those found in Alpha, Beta, Gamma and Delta variants, but Δ69–70 is unique for Alpha and Omicron variants [3, 5]. These mutations resulted in a gradually increased transmissibility and viral binding affinity, as well as higher antibody escape, higher viral load, longer duration of infectiveness, and higher rate of reinfection [2]. Among the 32 mutations of BA.1 on spike proteins, fifteen of them affect the receptor binding domain (RBD). BA.2 shares most of its mutations, but has 28 distinct ones. On the RBD, BA.2 has four unique mutations and 12 shared with BA.1. BA.3 shares most of its mutations with BA.1 and BA.2, except for one on NSP6 [2]. Binding free energy (BFE) charge between the S protein RBD and ACE2 receptor determines the virulence of variants: the larger the BFE charge is, the higher infectivity will be. Omicron variants BA.1, BA.2, and BA.3 have BFE charges of 2.60, 2.98, and 2.88 kcal/mol, respectively, which are much higher than those of other major variants, including the Delta variant. Vaccine breakthrough, evasion of antibody protection also depends on high BFE charge of Omicron subvariants that resulted in the ousting of Delta variant [2]. For the virus, it is an ongoing adaptation to the human host through accelerated phylogenesis. However, accumulation of the immense number of mutations can evolve through many replication cycles, several virus generations, and longer time. Although Omicron variants represent a new monophyletic clade that is distant from other variants, sequence alignment using the simplified Jukes-Cantor substitution model unambiguously proved their genetic relatedness to the Alpha variant. It is assumed that accumulation of mutations occurred during circulation of this coronavirus in chronically infected patient or patients, especially if they were immunocompromised with diminished capacity to eradicate infection [1].

## High prevalence of HIV infection in South Africa could predispose for SARS-CoV-2 infection

The Alpha variant (B.1.1.7) was isolated from a cancer patient in UK in September 2020. It emerged from the D614G variant [6, 7], whereas omicron BA.1 was obtained from an HIV-infected patient in Botswana, South Africa (SA), more than 1 year later [1]. This suggests that different forms of immunocompromised states of the individual might predispose to selection of coronavirus mutants.

The extremely high prevalence of HIV infection and AIDS in South Africa could have contributed to the emergence of the omicron variant [1]. This specific situation is dramatically different from those in other countries [3]. SA has a population of roughly 57 million people. Estimates suggest that 7.7 million individuals are living with HIV, representing approximately 14% of the population that equals 1 in 7 persons. The HIV prevalence among adults between 15 and 49 years is more than 20% (1 in 5 person), 13% of black citizens, whereas 0.3% of white citizens are HIV infected. Only below 70% of infected people are on antiviral therapy. On the contrary, the proportion of fully vaccinated population against SARS-CoV-2 is only 24%; this value is far lower than the average vaccination percentage of 42% globally [3]. Since early 2020, three big waves of COVID-19 outbrakes have been recorded in SA. Among them, two are caused by the Beta and Delta variants [3]. A longitudinal study conducted in KwaZulu-Natal, a province with one of the highest adult HIV prevalence in the world between 2004 and 2019, showed important changes in HIV endemic [8]. During this period, the median age of seroconversion increased by 5.5 years in men from age 25.5 to age 31, and by 3 years in women from 21 to 24 years. But HIV prevalence has more than double in those above 25 years of age. Mathematical models suggest that new HIV infections will become increasingly concentrated in older adults [8]. Another large study showed that in SA, HIV infection was associated with a doubling of COVID-19 mortality risk, independent of CD4 count or HIV viral load [9]. Individuals with immunocompromised conditions including HIV infection would be at particularly high risk of both SARS-CoV-2 acquisition and severe disease course. Contrary to former studies, that found no increased risk for SARS-CoV-2 infection, analysis of reports from many countries worldwide clearly indicates an increased risk for severe COVID-19 disease of people living with HIV (PLWH) even in settings of well-controlled HIV infection [10]. Throughout the first waves of SARS-CoV-2 pandemic, elderly people were primarily affected by the most severe clinical course of COVD-19 and died in highest proportions. In many aspects, AIDS is regarded as an extremely accelerated aging process [11, 12]. Despite the highly active antiretroviral therapy (HAART), life span of HIV-infected individuals is 10 years shorter as compared to healthy individuals [12]. Although HAART has significantly modified the natural course of AIDS, transforming it to a chronic disease, AIDS-related burden is replaced by aging-related complications [12]. Cells carrying integrated HIV cannot be eradicated from the body. Despite HAART, a small fraction of virus escapes from control, and promotes chronic inflammation [12], one of the key factors during development of severe respiratory disease [10]. The cellular immunity recovers to variable degrees, and such individuals remain at greater risk for many infections. PWLH frequently have overlapping risk factors for COVID-19, often at higher rates than the general population, e.g., being male, black or Hispanic, smoking, older age, and medical comorbidities such as hypertonia, dyslipidemia, type 2 diabetes mellitus, asthma, and chronic obstructive pulmonary diasese (COPD) [10]; many of them can also be regarded as age-related problems. In people, who are immunocompromised for different reasons, the SARS-CoV-2 persists for much longer time than in immunocompetent individuals [1]. As a paradox, immune reconstitution of HIV-infected patients could also exacerbate the inflammatory response and worsen respiratory failure upon SARS-CoV-2 infection [10]. Social factors of many PLWH, especially for those among low socio-economic status, as unstable living conditions, restricted access to health care systems can contribute to high prevalence of SARS-CoV-2 infection [references in 10]. The shift of primary HIV infection to older adults in SA, who might have several comorbidities, run down physical conditions and immune status, raised the chance for superinfection by an ancient version of SARS-CoV-2 virus. Several medically unattended HIV carrier individuals could harbor and transmit the descendant mutants of the old variant without revealing the exact causing factors of their worsening symptoms for a longer time, even, that would be regarded as progressive AIDS [3]. Large number of susceptible hosts, many of them in advanced or terminal stage of AIDS, in a geographical region predisposed for SARS-CoV-2 infection. To make possible sequence of events clear, HIV infection preceded coronavirus infection and paved the way for coronavirus to take advantage for its immense uncontrolled replication. Although the exact molecular interaction between SARS-CoV-2 and HIV is not known, the similarities in their replicative machinery and regulation might contribute to evolving Omicron variant.

## Unique role of ExoN in the life cycle of coronaviruses

High frequency of mutations is characteristic to RNA viruses due to lack of proofreading that results in low-fidelity replication. Diverse genome mutant populations coexist as “quasispecies.” By this special feature, RNA viruses can adapt to various replicative environments. Two families of *Nidovirales* (*Coronaviridae* and *Arteriviridae*) have unique replication strategy among RNA viruses. Very briefly, two thirds of the genome codes for 16 non-structural proteins designated from nsp1 to nsp16. They facilitate virus replication and production of structural proteins (nucleocapsid /N/, membrane/M/, envelope /E/, spike /S/). Transcription is mediated by nsp12, the RNA-dependent RNA polymerase (RdRp). Coronaviruses encode their own capping machinery to stabilize RNA. When this enzyme transfers a guanosine monophosphate to the 5′-end of the mRNA, subsequently, this guanosine is methylated by the nsp14-nsp10 heterodimer to generate N^7^-methylated Cap*-O*-RNA (m^7^-GpppA-RNA). The next step is to form the Cap-1-RNA; a methyl group is transferred from S-adenosylmethionine (SAM) to the 2′-OH of the first adenosine ribonucleotide. This reaction is catalyzed by the 2′-O-methytransferase (MTase), a heterodimer complex of nsp16 with the activator nsp10 [13]. In case of both SARS-CoVs and the Middle East respiratory syndrome coronavirus (MERS-CoV), methyl transfer is activated by divalent cations. SARS-CoV nsp14 is activated by Mn^2+^, but not by Mg^2+^, whereas nsp16 is activated by Mn^2+^ or Mg^2+^, as to a lesser extent by Ca^2+^ [14]. But nsp14 is a bifunctional, 60 kDa protein: the C-terminal N7-methyltransferase (N7-MTase) domain is involved in mRNA capping, and the N-terminal 3′ to 5′ exonuclease (ExoN) domain corrects errors made by RdRp by removing mismatched nucleotides from the 3′ end of the growing RNA strand [15]. Its efficacy is imperfect; its functional activity can repair 94% of mismatches. An infected individual might contain 10^11^ virions; consequently, several new mutants may be formed in every replication cycle [6]. ExoN activity requires preferably Mg^2+^ over Mn^2+^, Co^2+^ as cofactors; the presence of Zn^2+^ allows only residual activity, while catalysis is not supported by Ca^2+^, Ni^2+^, and Cu^2+^ [16]. ExoN has two zinc fingers that are essential for exonuclease function. ExoN is also involved in the maintenance of the largest genome (26 to 32 kb) among RNA viruses. No close orthologs of ExoN can be found in host cells [17]. Studies have demonstrated that knock out mutations of ExoN greatly reduce coronavirus transcription fidelity [6, 16]. SARS-CoV-2 binds membrane bound angiotensin-converting enzyme 2 (mACE2) that is a Zn^2+^ containing metallo-enzyme located on the surface of several types of cells, but lymphocytes lack this receptor [18]. As a summary, two protein complexes take part in mRNA methylation: namely, nsp14 with nsp10 activated by Mn^2+^ and nsp16 with nsp10 also activated by Mn^2+^ In contrast, ExoN activity prefers Mg^2+^. Selective inhibition of ExoN by disturbed Mg^2+^ homeostatsis could retain the ability of virus replication with a diminished proofreading, with the consequent emergence of a myriad of mutants, among which non-lethal ones also occur. Inhibition of the functions of other nsp gene products would interrupt virus replication. We propose a new hypothesis, that the selective inhibition of ExoN without affecting the MTase activities can be mediated by altered homeostasis, primarily by the practically dysregulated supply of Mg^2+^ cations.

## HIV coinfection may modulate divalent cation homeostasis

Throughout the first waves of SARS-CoV pandemic, elderly people were primarily affected by the most severe clinical course of COVID-19, and died in the highest proportions. In many aspects, AIDS is regarded as an extremely accelerated aging process [11]. Highly active antiretroviral therapy (HAART) has significantly modified the natural course of AIDS, transforming it to a chronic disease; however, cells carrying integrated HIV cannot be eradicated. In spite of HAART, a small virus fraction escapes from control and promotes chronic low-grade inflammation with elevated pro-inflammatory cytokine profile [11, 12].

In successive stages of HIV infection and AIDS, homeostatic alterations of divalent cations play a crucial role in the interactions between viral and host factors. Dietary supplementation of divalent cations enhances HIV-1 replication, whereas their chelation suppresses it and hinders AIDS progression [19]. Magnesium and manganese levels are elevated in oxidative stress promoting HIV-1 replication. HIV-1 reverse transcriptase (RT) and integrase as well as RNAse H enzyme activities are stimulated by magnesium and manganese. HIV regulatory gene products (Tat, Vif, Nef, Rev) also require divalent cations for their activity. Binding Tat to cellular cyclin T1 to promote HIV transcription is under the control of zinc. For people living with HIV (PLWH), serum levels of Zn^2+^ decrease with disease progression, and are associated with higher mortality rates. Zn^2+^ is a cofactor for HIV integrase, nucleocapsid, Tat, and Vif proteins. Evidence suggests that Mg^2+^, Mn^2+^, and Zn^2+^ act in a concerted manner [19]. Ferrous iron (Fe^2+^) controls various cellular functions. It is required in HIV-1 transcription, packaging, and RT activity. HIV-1 Nef and Rev polypeptides alter iron homeostasis. Copper has essential functions in the cellular anti-oxidative system, but exerts anti-microbial properties, among them inhibits HIV-1 protease activity. Selenium is essential for the cellular antioxidant mechanisms and immune functions. With lower levels of Se^2+^, PLWH have elevated levels of oxidative stress, higher viral load, increased opportunistic infections and higher mortality rates [see references in 19].

HIV unequivocally utilizes cations, thus altering the cellular ion homeostasis. Beside abnormal cytokine and chemokine patterns induced by HIV, altering ion exchange mechanisms can be an additional way to modulate functions of several [20]. These two viruses replicate simultaneously in the same organism infecting distinct cell types. HIV species do, coronaviruses do not replicate in lymphocytes. It is conceivable that chronic HIV infection results in gradual deterioration in the functions of more types or cells, as compared to effects of acutely replicating coronaviruses. Saying again, among many existing pathological alterations of HIV-infected patients, coronaviruses encounter with previously altered homeostasis of divalent cations during their replication.

Divalent cations play a role in regulating cellular functions involving dependent metalloproteins and metalloenzymes. Divalent cation accumulation has been implicated in aging process, immune reactions, and pathological conditions among them infections [19]. The intracellular level of the ionized, free cytosolic Ca^2+^ represents a characteristic signal transduction mechanism via rapid mobilization across plasma membrane from and to the extracellular space. The alkaline earth metal Ca^2+^ and transition metal Zn^2+^ have a 10^4^-fold concentration gradient between the cytosolic and extracellular compartments, while Mg^2+^ is only characterized by a < twofold gradient. Deficiency in cytosolic free Mg^2+^ results in low CD4^+^ T cell number, decreased antibody production, chronic inflammation, and promotes the release of pro-inflammatory cytokines such as IL-1β, TNF-α, and IL-6 [20]. All of these phenomena are characteristic to AIDS patients. Cytosolic free Zn^2+^ is maintained at low levels by tight regulation. Zn^2+^ deficiency decreases T cell activation, causes a shift from Th1 response towards Th2 response, and alters IL-6 mediated pathways [20]. The net effect of these changes can result in a relative Mg^2+^ starvation in coronavirus-infected cells consequently diminishing activity of its ExoN enzyme.

There is a logical approach whether antiretroviral therapy (ART) can normalize divalent cation levels and render HIV-infected persons more resistant to SARS-CoV-2. HAART and micronutrient supply can modulate the level and activity of certain divalent cations, but reports are scarce and incomplete. HIV RT is one of the major targets for nucleoside (NRTI) and non-nucleoside (NNRTI) RT inhibitors. As mentioned above, its activities require divalent cations. At physiological cellular Mg^2+^ concentration (0.25 mM), the primer-extension activity is moderate, but the fidelity of nucleotide pairing is increased several-fold. Mg^2+^ concentrations alter the potency of RT inhibitors. Inside cells NRTIs show less inhibitory activity than in high level (6 mM) in vitro tests. The RT inhibitory activity of a new drug candidate, the “translocation-defective RT inhibitor” (EFdA, 4 ethynyl-2 fluoro-2′-deoxyadenosine), is unaffected by Mg^2+^ concentration [21]. NNRTIs and divalent cation-dNTP (deoxyribonucleotide triphosphate) complexes compete with each other for binding to RT. Mn^2+^, Zn^2+^, and Co^2+^ containing complexes resulted in a stronger resistance to NNRTIs than Mg^2+^ and Zn^2+^ containing complexes. The first generation NNRTI drugs (efavirenz, EFV, nevirapine, NVP, delavirdine, DLV) are less competitive with the divalent cation-NTP complexes than the second generation drugs (rilpivirine, RPV, etravirine, ETV) [22]. Micronutrient supply also is common among HIV-infected individuals with or without HAART. Beside other compounds not mentioned here, studies on their effect on serum divalent cations show conflicting results. A study in the USA showed that before the HAART era, three quarters of HIV infected were selenium deficient, on quarter was zinc deficient. After HAART, 10% remained Se^2+^ deficient, but the ratio of Zn^2+^ deficiency remained the same. Se^2+^ and Zn^2+^ intake had no effect on their serum levels. It is concluded that low cation levels could be by viral utilization and sequestration in proteins. Improved virological control with HAART could result in the higher Se^2+^ levels [23]. A randomized controlled trial in HIV-positive pregnant or lactating women in Tanzania was conducted, who did not take antiretroviral treatment. Subjects got Zn^2+^ or Se ^2+^ or placebo. No significant clinical benefits were found in the micronutrient receiving groups as compared to the placebo treated group. Treatments did not delay HIV disease progression or improve pregnancy outcomes [24]. This result suggests that only HAART treatment can compete with the HIV-induced pathological alterations of divalent cation activity, but the effects are not exclusive. Micronutrient treatment, regarding at least divalent cations, cannot restore their activity to physiological level. This type of practice widely applied in poorer countries cannot replace HAART treatment. One can conclude that although not completely, but partially HAART treatment could decrease the susceptibility of HIV-infected individuals to severe coronavirus disease.

## Lessons from the interaction of HIV with heterologous viruses

Interactions of different viruses within the same host are not uncommon leading to genetic and non-genetic relationships [25]. A virus can hijack a gene from another virus if both replicate in the same cell: e.g., human herpesvirus/HHV/-6 obtained its U94 gene from adeno-associated virus (AAV/-2) [26]. Both HHV-6 U94 and AAV-2 *rep* genes inhibit transcription of other viruses among them HIV-1 and repress the expression of cell oncogenes [27]. Replication of defective animal retroviruses depends on a replication-competent retrovirus. Different mutants of the same virus can complement the defective enzymatic functions of each other [28]. However, simultaneous infection of the same cell is a relatively rare event in vivo. Viruses replicating in different cells can modulate the replication of each other and the pathological sequale caused by them through soluble mediators. This scenario is biologically more frequent and relevant. Such viral interactions gained considerable attention with the discovery of HIV. Several microbes elicit opportunistic infections in AIDS patients with a consequence of the chronic impairment of cellular immunity. Heterologous viruses, such as adenoviruses, herpesviruses, hepatitis D virus, and leukemia viruses, augment HIV replication resulting in the acceleration of AIDS progression. These interactions were also proven in the feline AIDS mode [25]. These DNA viruses harbor early or immediate early genes; retroviruses possess regulatory genes, whose expression not only augment their own replication, but through activating cellular transcription factors, activate HIV or FIV. RNA viruses, including coronaviruses, do not encode such genetic elements; they do not activate lentiviruses directly [25]. Early in vitro experiments indicated that transactivation could take place in the same cell, but later both epidemiological surveys and model experiments helped the identification of the exact mechanism of transcellular transactivation between different infected cells [25]. Abnormal cytokine and chemokine patterns released from cells infected with heterologous viruses induce pathological activation of transcription factors in HIV carrier cells. These factors bind to HIV long terminal repeat (LTR) enormously increasing its replication. Transactivation and opportunistic infections collectively cause a vitious circle [25]. However, less attention was devoted to transactivating viruses. Several types of adenoviruses can simultaneously infect cells in immunocompromised host, or reactivate from latent state, replicate in the same cells, resulting in recombination. From the stool of AIDS patients, both recombinants and 9 new types of adenoviruses have been identified; furthermore, from their urine, several intertypic recombinants of B species have been obtained. Such recombinants are not found in immunocompetent individuals [29]. Adenovirus infection rate in AIDS patients is higher than that in organ recipients suggesting that type and severity of immunosuppression might predispose to different range of opportunistic infections [29]. Studies performed in North America and Europe showed that prevalence of acyclovir resistant herpes simplex virus (HSV) mutants in HIV-infected patients was relatively frequent, between 4 and 7% [30] or 3.6 and 10.9% [14]. In contrast, resistant HSV spontaneously emerges in immunocompetent hosts between 10^−4^ and 10^−5^ frequency, and their presence is transient [31]. At the time of observations, the molecular mechanisms, how HIV can contribute to the emergence of adenovirus recombinants and resistant HSV mutants, was not studied [29, 31]. It was presumed that in patients with deficient T cell immunity, extensive and prolonged viral replication, without the selection of thymidine kinase-deficient herpesviruses could provide an ideal scenario for the shedding and transmission into the population [30]. Such phenomena are not characteristic to immunocompetent individuals. This clearly suggest, that, besides continuously providing a diminished immunological background, HIV must exert further, more targeted molecular influence on the replication machinery of heterologous viruses, including SARS-CoV-2.

Further studies to elucidate the mechanism of emergence of mutant heterologous viruses in HIV-infected patients are clearly warranted. Based on South African epidemiological and international molecular studies, it is forecasted that Omicron subvariant BA.2 might initiate the next COVID-19 pandemic [2] allowing enough time to take worldwide precautions against this highly deadly disease. Hopefully, there will be sufficient interest and support to study the exact mode of emergence of coronavirus mutants. Inhibition of such biological processes could clearly be a successful element of a preventive strategy. In those poor countries, where HAART of AIDS and vaccination against SARS-CoV-2 is not widely available, both measures may be implemented. Experience from SA shows that younger people benefit from increasing HAART coverage, medical interventions, such as voluntary male circumcision to avoid contracting HIV infection [8] and, consequently, coronavirus superinfection.

## Conclusions

Isolation of the mutation-rich Alpha variant from a cancer patient [7] and recently the Omicron variant from an HIV-infected patient [1] raises the idea that preceding severe immunocompromised state of the host might predispose to the selection of SARS-CoV-2 mutants. We propose a hypothesis to elucidate this phenomenon. SARS-CoV enzymes and the HIV replication machinery require the same divalent cations. Coronavirus likely encounters severely altered ion homeostasis in HIV-infected hosts including the relative disturbance of Mg^2+^ level. Selective inhibition of Mg^2+^-dependent ExoN activity independently from Mn^2+^-dependent activities of nsp14 and nsp16 complexes with nsp10 may result in the gradual accumulation of mutations through several replication cycles in HIV-infected hosts. Clinical and experimental studies on HIV activating heterologous viruses suggest that transcellular alterations in the metabolism of ACE2 carrying cells are mediated by abnormal interleukin production of HIV-infected lymphocytes. Viral persistence and immune evasion by the SARS-CoV-2 mutants increase their survival and selection, as an example of coronavirus phylogenesis. NNRTI drugs but not micronutriments partially restore Mg^2+^ homeostasis indicating that HAART of HIV-infected individuals decrease their susceptibility to SARS-CoV-2 infection and emergence of mutants.

## Abbreviations

AIDS: Acquired immune deficiency syndrome
ART: Antiretroviral treatment
BFE: Binding free energy
COVID-19: Coronavirus disease-19
COPD: Chronic obstructive pulmonary disease
DLV: Delavirdine
dNTP: Deoxyribonucleotide triphosphate
FIV: Feline immunodeficiency virus
EFdA: 4 ethylnyl-2 fluoro-2’-deoxyadenosine
EFV: Efiravenz
ETR: Etravirine
ExoN: N-terminal exonuclease
HAART: Highly active anti-retroviral therapy
HIV: Human immunodeficiency virus
HSV: Herpes simplex virus
LTR: Long terminal repeat
mACE2: Membrane bound angiotensin converting enzyme 2
MERS: Middle East respiratory syndrome
MTase: Methyltransferase
Nef: Negative regulating factor
nsp: Non-structural polypeptide
NTD: N terminal domain
NVR: Nevirapine
nsp: Non-structural protein
N7-MTase: N7- methyltransferase
NNRTI: Non-nucleoside reverse transcriptase inhibitor
NRTI: Nucleoside reverse transcriptase inhibitor
NTD: N-terminal domain
PLWH: People living with HIV
RdRp: RNA dependent RNA polymerase
Rev: RNA splicing regulator
RPV: Rilpivirine
RT: Reverse transcriptase
S: Spike
SA: South Africa
SAM: S-adenosyl methyltransferase
SARS-CoV-2: Severe acute respiratory syndrome-coronavirus-2
Tat: Transactivator protein
Vif: Viral infectivity factor
VoC: Variant of concern
WHO: World Health Organization
